# Energy carbon emission structure and reduction potential focused on the supply-side and demand-side

**DOI:** 10.1371/journal.pone.0239634

**Published:** 2020-10-06

**Authors:** Jijun Kang, Yanjun Yang

**Affiliations:** School of Economics and Business Administration, Chongqing University, Chongqing, China; Institute for Advanced Sustainability Studies, GERMANY

## Abstract

In recent years, the environmental problems caused by excessive carbon emissions from energy sources have become increasingly serious, which not only aggravates the climate change caused by the greenhouse effect but also seriously restricts the sustainable development of Chinese economy. An attempt is made in this paper to use energy consumption method and input-output method to study the carbon emission structure of China's energy system and industry in 2015 from two perspectives, namely China's energy supply side and energy demand side, by taking into account the two factors of energy invest in gross capital formation and export. The results show that neglecting these two factors will lead to underestimation of intermediate use carbon emissions and overestimation of final use carbon emissions. On energy supply side, the carbon emission structure of China's energy system is still dominated by high-carbon energy (raw coal, coke, diesel, and fuel oil, etc.), accounting for more than 70% of total energy carbon emissions; on the contrary, the natural gas such as clean energy accounts for only 3.45% of total energy carbon emissions, indicating that the energy consumption structure optimization and emission reduction gap of China's energy supply side are still substantial. On energy demand side, the final use (direct consumption by residents and government) produces less carbon emissions, while the intermediate use (production by enterprises) produces more than 90% of the total energy carbon emissions. Fossil energy, power sector, heavy industry, chemical industry, and transportation belong to industries with larger carbon emissions and lower carbon emission efficiency, while agriculture, construction, light industry, and service belong to industries with fewer carbon emissions and higher carbon emission efficiency. This means that the optimization of industrial structure is conducive to slowing down the growth of energy carbon emissions on the demand side.

## Introduction

The growth of the world economy has not only resulted in the sharp increase in energy consumption, but also has aggravated environmental pollution and global warming. Especially since the 21st century, the global warming caused by excessive emissions of carbon and other greenhouse gases has become increasingly prominent. Since China is currently the country with largest carbon emissions in the world, many countries regard China as the destination of "carbon leakage" and put pressure on the Chinese government in the international climate negotiations [1]. In addition, for China, the dilemma between economic development and environmental pollution has become prominent. Therefore, whether it is the pressure of international public opinion or the need for internal sustainable development, China is required to take the responsibility of carbon emission reduction actively.

To reduce carbon emissions, the Chinese government has actively cooperated with other countries and international environmental protection organizations. In 2009, the Chinese government officially announced at the Copenhagen climate conference that the carbon emission intensity of China's GDP by 2020 will be about 40% to 45% lower than that in 2005 [2]. In 2014, the Chinese government made a commitment at the APEC meeting to strive to reduce carbon emissions per unit of GDP by about 60% to 65% in 2030 compared to the figure in 2005. In 2016, the Chinese government formally joined the "Paris Climate Change Agreement" and promised to work with other member states to cope with the problems caused by climate change. In the domestic, the Chinese government promulgated the 12th Five Year Plan for national economic and social development in 2011, which requires reducing greenhouse gas emission intensity. In 2016, China promulgated the outline of the 13th five-year plan for national economic and social development, which proposed that China should actively control carbon emissions and implement emission reduction commitments. The above commitments of the Chinese government has not only shown China's determination to reduce emissions, but also the objective and inevitable requirement of sustainable economic and social development.

To achieve the goal of emission reduction, the most important task of the Chinese government is to determine the actual foothold of emission reduction. Previous studies have suggested that the supply side and demand side factors should be considered in the calculation and analysis of China's carbon emissions [3]. Carbon emissions on the supply side mainly include energy carbon emissions [4], carbon emissions during cement production [5], and biomass decomposition carbon emissions [6]. According to statistics, China's carbon emissions mainly come from energy carbon emissions [7]. For demand side carbon emissions, it mainly includes intermediate use carbon emissions and final use carbon emissions [8]. Among them, the final use carbon emission refers to the carbon emission generated by residents and government consumption [9]; the intermediate use carbon emission refers to the carbon emission generated by enterprise consumption, and this part of carbon emission is the main component of carbon emission in consumption category [10]. To sum up, the foothold of China's emission reduction is the energy supply side and the industrial system on the demand side.

At present, the energy consumption method can be used to study the carbon emission of the supply side energy system, and the input-output method can be used to study the carbon emission of the industrial system on the demand side [11]. It should be noted that when using the input-output method, there will be some implicit factors affecting the calculation results of demand side energy carbon emissions, such as energy invest in gross capital formation and export. Among them, energy invest in gross capital formation refer to the part of energy used as inventory and fixed capital formation [12]. If this part of energy is not burned, it will not produce carbon emissions; energy export refers to the part of energy products exported to foreign countries or transferred out to local areas [13]. The combustion process of this part of energy is not local, and the carbon emitted is not a local emission. Therefore, in order to more accurately measure the carbon emissions of energy supply-side and demand-side industrial systems, the above two factors must also be considered. On this basis, considering the energy invest in gross capital formation and export, this paper uses the energy consumption method and input-output method to analyze the carbon emissions of the energy supply side and the demand side, so as to provide a more accurate policy basis for the Chinese government to achieve energy conservation and emission reduction.

The remainder of this paper is as follows. The second part is the literature review. The theoretical and scientific conceptual model is proposed with the relevant data in third part. The fourth part analyzes the Chinese industrial and energy system carbon emission structure. The conclusions and policy recommendations are presented in the last part.

## Literature review

According to the research theme of this paper, the literature review includes studies on energy supply side carbon emission, energy demand side carbon emission, and studies considering both supply side and demand side carbon emission.

### The carbon emissions on the energy supply-side

This carbon emission refers to the emissions generated by the final consumption of various forms of energy produced in the energy system. Xu et al. [14] analyzed the carbon emission factors of China's on the supply side and found that the energy consumption structure has an important impact on carbon emission. Long et al. [15] found that coal consumption played a leading role in carbon emissions. Zhang and Chen [16] used the neural network method to predict China's coal consumption and carbon emissions and concluded that China's coal consumption and carbon emissions would maintain a relatively stable growth trend in the future. Yu [17] use the economy -cost of carbon emissions (ECC) multi-objective optimization model to predict the carbon emissions peak, the results show that China's energy carbon emissions will reach peak between 2025 and 2028, during this period, if the gross domestic product (GDP) to maintain an annual growth of 5.9% to 6.3%, carbon emissions of the average annual growth rate will reach 0.5% 1.1%. Zhao and Luo [18] used the Vector Error Correction Model (VECM) to study the relationship between carbon emission intensity and coal and crude oil consumption and predicted China’s future energy consumption structure and carbon emissions. The results of the study showed that the energy consumption structure optimization will not only reduce the carbon emission intensity, but also reduce the GDP growth rate. With the development of renewable energy technologies, scholars have also conducted research on the relationship between renewable energy consumption and carbon emissions. The study found that the consumption of renewable energy reduced environmental pollution [19]. Nguyen and Kakinaka [20] found that in low income countries, renewable energy consumption was positively correlated with carbon emissions. In high income countries, renewable energy consumption is negatively correlated with carbon emissions.

### The carbon emissions on the energy demand-side

Since the energy consumption on the demand side mainly comes from the intermediate use of the enterprise, the research based on the demand side mainly focuses on the carbon emission of the production process of the enterprise in different industries [21, 22]. Among them, the construction, power, cement is large in scale, and these industries are energy-intensive, so the carbon emissions of these industries have particularly attracted the attention of more scholars [23]. Chen [24] used the input-output method to calculate the implied carbon emissions in the industrial production process and found that China's construction industry was the highest implied carbon emissions among all the secondary industries in 2002. Yang and Lin [25] used logarithmic exponential decomposition method to study the carbon emissions of China's power industry and pointed out that power intensity and economic activity were the main driving factors affecting the growth of carbon emissions. Wang et al. [26] calculated the carbon emissions of China's transportation infrastructure industry and found that more than 80% of the carbon emissions of expressway projects came from the production of raw materials, while site construction and material transportation only accounted for 10% and 3% of the total carbon emissions respectively. Tian et al. [27] found that the spatial distribution of agricultural carbon emissions in China is unbalanced, and this spatial disequilibrium feature shows an increasing trend. Yan et al. [28] analyzed the variation characteristics of agricultural carbon emission intensity in China, and the results has shown that agricultural carbon emission intensity showed a trend of steady decline. Lin and Lei [29] found that energy intensity is an important factor affecting the increase or decrease of carbon footprint of China's food industry. Wang et al. [30] calculated and analyzed the carbon emissions of China's service industry from 1995 to 2010, and found that China's service industry energy consumption is mainly dependent on high-carbon energy sources such as oil and coal, so the overall carbon emissions of China's service industry are on the rise. Tang et al. [31] proposed a factorization model to analyze the carbon emissions of China's tourism industry and found that the expansion of tourism scale and the growth of tourism output are the main reasons for the increase of carbon emissions of tourism industry.

### Both the supply side and the demand side are considered

Peng et al. [3] used the SDA method to investigate the factors affecting China's production and consumption side carbon emissions, the result showed that between 1995 and 2009, the production side and consumer side carbon emissions are increased significantly, and the growing rate of the production side is greater than the consumption side, structural decomposition results show that the production side and consumer side the cause of the rapid growth of carbon emissions is the size of the final demand growth and investment structure, production department and production department the decrease in the strength of carbon emissions effectively suppresses the production side and consumer side of carbon emissions, but the inhibition on the wane. Wang et al. [32] established a hybrid energy model based on energy demand and energy supply balance to predict the structure and carbon emission trend of China's future supply side energy route, they found that China is still unable to get rid of the dependence on fossil fuels, and nuclear power after 2020 is expected to become the main alternative to fossil fuel. Due to the constraints of the current energy consumption structure, China's carbon emissions will reach the peak in 2025.

Overall, most researches only focus on the supply side of the energy consumption or demand side impact on carbon emissions, which is unable to fully investigate the impact of higher energy consumption on carbon emissions. Although a few literatures consider the impact of energy consumption on carbon emissions at the same time, they do not consider the impact of energy invest in gross capital formation and export on carbon emissions.

### The academic contribution of this paper

Compared with the previous research, there are two innovative points in this paper. Firstly, based on the consideration of energy invest in gross capital formation and export, this paper unifies the supply side and demand side of energy consumption to study the impact of energy consumption on carbon emissions. Secondly, this paper analyzes the structural characteristics and efficiency differences of carbon emissions on the energy supply side and the industrial demand side.

## Data and model

### Data

This paper chooses China's energy CO_2_ emission in 2015 as a case and analyzes the CO_2_ emission structure of China's energy supply-side and demand-side in 2015. The selected data sources mainly included China's energy balance sheet and input-output table in 2015, the average low heating value of each energy source and CO_2_ emission coefficients in 2015.

### Method and calculation

This paper uses energy consumption method and input-output analysis to analyze the structure of China's energy carbon emission. For detail, we employ the energy consumption method to calculate the energy carbon emission and analyze the structure of energy carbon emissions on the energy supply-side. Meanwhile, we also use the input-output analysis to calculate the carbon emission from industry and analyze the structure of carbon emissions on the energy demand-side. After that, this paper analyzes the impact of energy consumption on carbon emissions on the basis of considering the two factors of energy invest in gross capital formation and export. The detailed calculation and analysis process is as follows:

Firstly, we can use the energy consumption method to calculate the CO_2_ emissions from energy combustion. When the CO_2_ emission coefficient is W and the amount of burned energy is B, the CO_2_ emission E is calculated as the following equation.

E=B×W (1)

Where W is CO_2_ emission coefficient, B is the amount of energy for combustion.

Due to different carbon emission coefficients for different energy sources (There are 20 energy sources in the China Energy Balance Sheet for 2015), the Eq (1) need to be modified. Assuming that the CO_2_ emission from burning energy *i* is the *E*_*i*_; energy consumption for burning energy *i* is the *B*_*i*_; CO_2_ emission factor of energy *i* is the *W*_*i*_. The CO_2_ emission of energy *i* can be computed by the following Eq (2).

$$E=\sum_{i}E_{i}=\sum_{i}B_{i}\times W_{i}$$ (2)

We should subtract the non-burning energy consumption (i.e. not used for combustion) from total energy consumption to get the energy consumption for combustion. According to the Energy Balance Sheet for 2015, the burning energy consumption includes the final energy consumption, the energy consumption used for thermal power generation, and the energy consumption used for heating. We should also subtract the energy consumption used for the industrial raw materials because the energy consumption used for the raw material belongs to the non-burning energy consumption. The total burning energy *i* is *B*_*i*_, the calculation process of *B*_*i*_ is as follow:

$$B_{i}=B_{i}^{T}+B_{i}^{P}+B_{i}^{H}-B_{i}^{M}$$ (3)

where the amount of energy source *i* consumption used for the final consumption is the $B_{i}^{T}$; the amount of energy source *i* consumption used for generating thermal power is the $B_{i}^{P}$; the amount of energy source *i* consumption used for heating is the $B_{i}^{H}$; the amount of energy source *i* consumption used for the raw materials in the industry. Then we can use the average lower heating value and the CO_2_ emission coefficient per unit heat to compute the CO_2_ emission coefficient of each type of energy for combustion. According to the China energy statistical Yearbook 2016, we can obtain the average lower heating value and the CO_2_ emission coefficient per unit heat of energy *i*. The CO_2_ emission coefficient of energy *i* for combustion can be calculated by multiplying these two factors together, and the coefficient of energy source *i* can be calculated as the following equation.

$$W_{i}=T_{i}\times Q_{i}$$ (4)

where the CO_2_ emission coefficient per unit heat from the energy *i* for combustion is the *T*_*i*_; the average lower heating value of energy *i* for combustion is the *Q*_*i*_. According to Eq (4), we can obtain the CO_2_ emission coefficient of energy *i* for combustion.

Secondly, we use the input-output method to calculate the industrial CO_2_ emission and analyze of the structure of industrial carbon emission on the energy demand-side. In the input-output table of China in 2015, China's industry is divided into 42 sectors. There are 4 energy sectors in 42 sectors. The relationship between these four energy sectors and various energy sources is shown in Table 1 below.

**Table 1:** The relationship between these four energy sectors and energy sources.

Energy industry	The coal mining industry	The petroleum and natural gas exploitation industry	The petroleum, cooking and nuclear fuel products industry	The gas production and supply industry
Categories of energy	The raw coal, clean coal and other washed coal	The crude oil and natural gas	The gasoline, kerosene, diesel, fuel oil, liquefied petroleum gas, refinery dry gas, coke, and other cooking or petroleum sources	The coke oven gas and other types of gas

According to the above analysis, it can be concluded that the carbon emissions from energy combustion produced by the four energy industries in China are the carbon emissions of the energy demand-side. The CO_2_ emissions of each sector is mainly calculated by evaluating the total amount of CO_2_ emissions from energy combustion according to its proportion of energy consumption.

The measurement error of energy carbon emissions will appear if the energy exported and invested in the gross capital formation is not taken into account. Without regard to the energy exported and invested in the gross capital formation, the total energy investment for the combustion of all energy industries is calculated vas following equation.

$$D_{j}=DI_{j}+DF_{j}$$ (5)

Where *D*_*j*_ is the total energy investment for the combustion of the energy industry *j*; *DI*_*j*_ is the intermediate demand energy for the combustion of the energy industry *j*, and *DF*_*j*_ is the final demand energy for the combustion of the energy industry *j*. If the two factors are subtracted, then *D*_*j*_ is computed as follows.

$$D_{j}=DI_{j}+DF_{j}‐DC_{j}-DE_{j}$$ (6)

Where *DC*_*j*_ the energy is invested in the gross capital formation from the energy industry *j*; *DE*_*j*_ is the energy exported from energy industry j. Then, the carbon dioxide emission coefficient of the energy industry *j* is measured according to Eq (7).

$$e_{j}=\frac{E_{j}}{D_{j}}$$ (7)

Where *e*_*j*_ is the CO_2_ emission coefficient of the energy industry *j*; *E*_*j*_ is the CO_2_ emission of the energy industry *j*. Using Eq (7), we can calculate the sectoral CO_2_ emission from energy combustion in the 42 sectors listed the input-output table. Based on Eq (8), the CO_2_ emission derived from energy industry *j* and generated from sector *k* can be calculated. According to Eq (8), we can simply achieve this by multiplying the generated energy investment in the energy industry *j* used for industry *k* and coefficient (for the industry *j*) that is computed by Eq (7). Thus, the total CO_2_ emission of the industry *k* includes the sum of the energy and the segment used for industry *k*, derived from each energy industry, which can be computed as follows.

$$E^{k}=\sum_{j}E_{j}^{k}=\sum_{j}B_{j}^{k}\times e_{j}$$ (8)

Where *E*^k^ is the CO_2_ emission of industry *k*; $E_{j}^{k}$ is the energy industry *j* derived CO_2_ emission which is generated from the industry *k*; and $B_{j}^{k}$ is the energy industry *j* generated energy investment which is used for industry k. According to the above equations, we can compute the sectoral CO_2_ emissions of the 42 sectors in China due to energy combustion. Then we divided the CO_2_ emissions of the sector *k* by the total output of the sector *k*.

$$f^{k}=\frac{E^{k}}{T^{k}}$$ (9)

Where *E*^k^ the CO_2_ emission of sector is *k*, *T*^*k*^ is the output of sector *k*, and *f*^*k*^ is the CO_2_ efficiency of the industry *k*. In this work, we merged 42 sectors into ten sectors.

## Results and analysis

### Impact of energy invest in gross capital formation and export on energy carbon emission measurement

In this study, there are two in influencing factors (energy invested in gross capital formation and energy exported), and 4 scenarios were designed. The details of the 4 scenarios are shown in Table 2.

**Table 2:** Scenario design and description.

Scenario	If energy invested in the gross capital formation is subtracted (1 is yes, and 0 is no)	If energy is exported (1 is yes, and 0 is no)
I	0	0
II	0	1
III	1	0
IV	1	1

**Fig 1:**
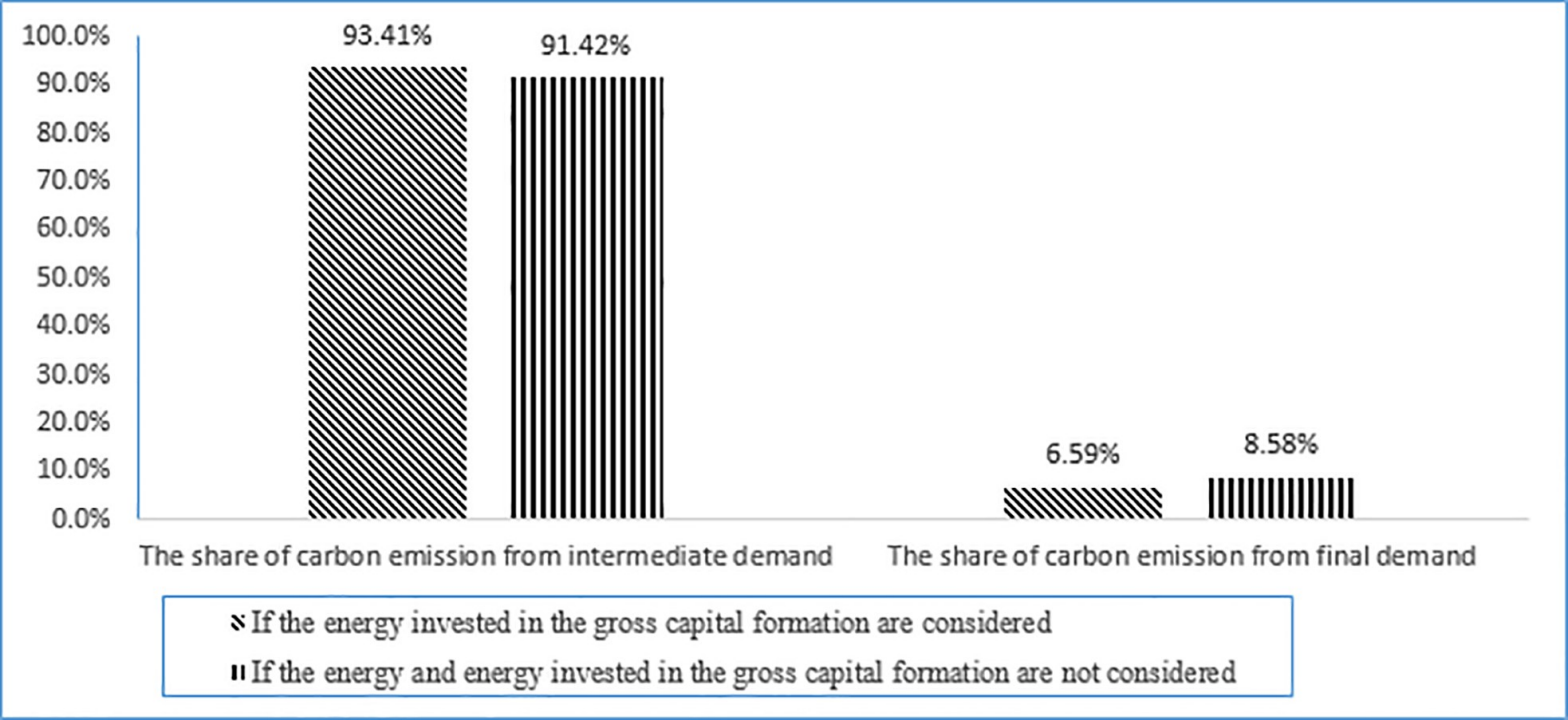
Calculation error of energy carbon emission when energy invest in gross capital formation and export are ignored.

This study started with the following two issues: "whether to subtract energy invest in gross capital formation" and "whether to subtract export and transfer energy input"; and considered that calculation error of energy carbon emission occurs when these two factors are ignored. Based on the aforementioned models and data, the carbon emission from intermediate and final demands under the two scenarios were calculated. The detailed results of carbon emission are shown in Table 3, and the structure of carbon emission is shown in Fig 1.

**Table 3:** Detailed results of carbon emission under two scenarios.

Structure of carbon emission	Carbon emission from intermediate demand	Carbon emission from final demand	Total carbon emission
If the energy invested in the gross capital formation are considered	1055692.850	74505.273	1130198.123
If the energy and energy invested in the gross capital formation are not considered	1033211.391	96986.732	1130198.123

By analyzing Table 3 and Fig 1, we can draw two conclusions. First, the total carbon emissions are not affected by energy invest in gross capital formation and export. This was because the total energy carbon emission is calculated according to two parameters (energy consumption and energy carbon emission coefficient), while energy capital deposit and loan and energy export have no direct impact on these two parameters. Second, if energy invest in gross capital formation and export are ignored, the total input amount of energy industry in formula 6 will be overestimated, thus the carbon emission coefficient of unit energy input in formula 7 will be underestimated, and the amount of carbon dioxide calculated by the industry (intermediate use) will also be underestimated; when the total carbon emission remains unchanged, it will lead to final use carbon emissions are overvalued. The reason for the above error is that the energy capital deposit and loan is kept in the form of fixed capital, which will not be burned and will not produce carbon emissions; the energy export is transferred out in the form of energy as a commodity, no matter whether it is burned or not, the carbon emission generated does not belong to the local government.

### Analysis of carbon-emission composition on the energy supply-side

The analysis of the structure of carbon emissions on the energy supply-side refers to the calculation of various types of carbon emissions on the energy supply-side. The results of carbon emissions from various energy sources in 2015 are shown in Table 4.

**Table 4:** CO_2_ emissions from energy consumption.

Categories of energy	Carbon emissions(10 kilo-tons)	Percentage
Raw Coal	688020.2911	60.88%
Coke	128731.4305	11.39%
Other Gas	112536.2332	9.96%
Diesel Oil	54618.244	4.83%
Natural Gas	38956.9704	3.45%
Gasoline	34245.7064	3.03%
Cleaned Coal	13622.6496	1.21%
Other Washed Coal	10886.0801	0.96%
LPG	10600.2658	0.94%
Kerosene	8242.7032	0.73%
Fuel Oil	7083.7522	0.63%
Other Petroleum Products	6278.1217	0.56%
Coke Oven Gas	5974.1613	0.53%
Refinery Gas	4573.268	0.41%
Other Coking Products	2232.8294	0.20%
Crude Oil	2041.9083	0.18%
Briquettes	1553.5082	0.14%

As can be seen from Table 4, the carbon emission coefficient of raw coal is relatively high, accounting for 60.88% of China's total emissions from energy consumption, while the carbon emission ratio of raw coal, coke, diesel, and other fuel oil is 87.06%. In contrast, the clean energy sources such as natural gas and liquefied natural gas accounted for a small part. This is because carbon-rich energy sources, including raw coal, coke, diesel and other fuel oils with high carbon emission coefficients, were still being vastly used while carbon-poor energy sources, such as natural gas and liquefied natural gas with low carbon emission coefficients, were far less consumed in China. The above results indicate that the carbon emissions in China were highly related to the composition of energy consumption on the supply-side, and there is still substantial room for optimization of the composition.

**Fig 2:**
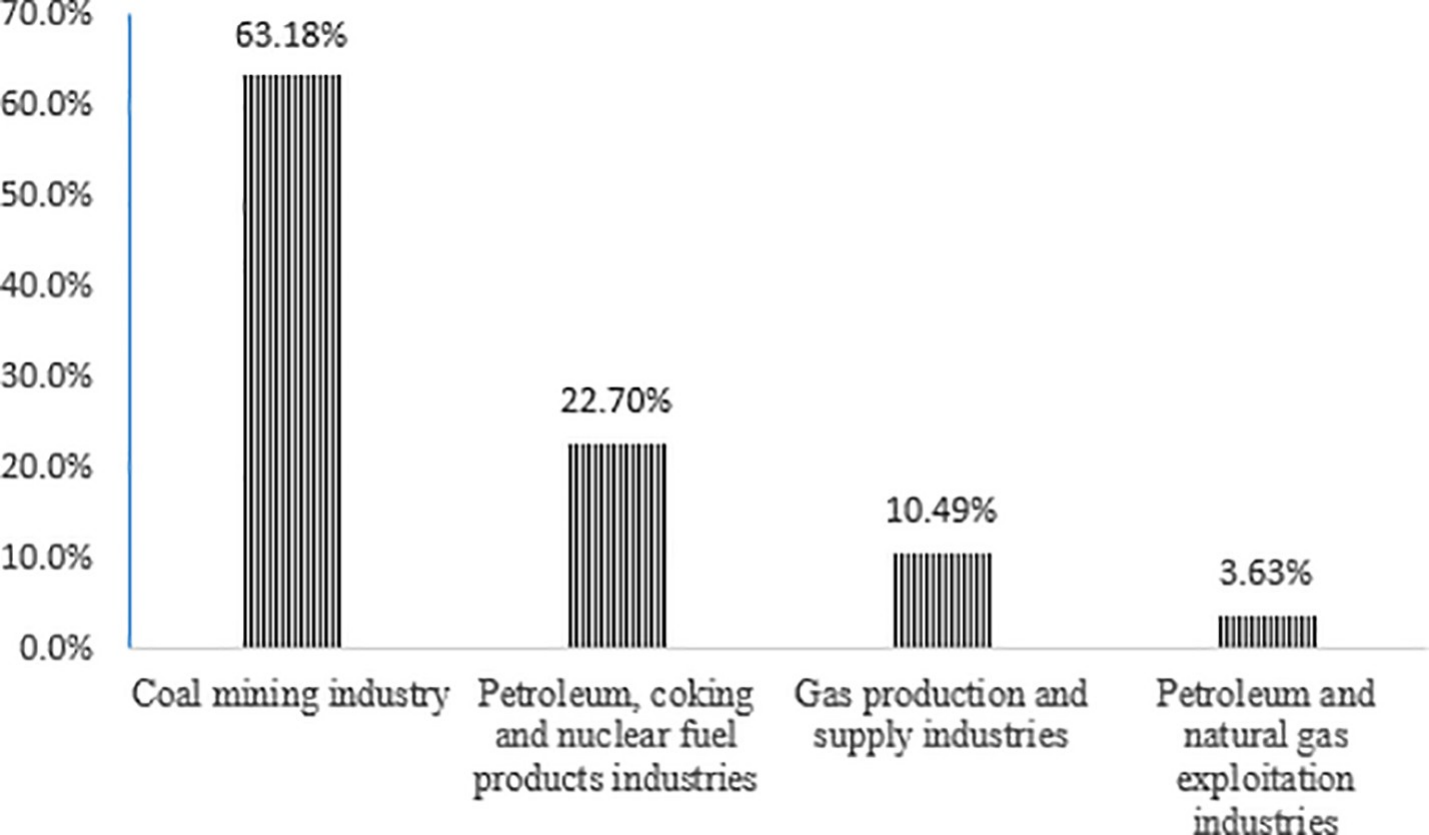
Proportion of carbon emissions from four types of disposable energy.

Moreover, four types of primary energy providers were further investigated to acquire the corresponding carbon emissions structure (Fig 2) caused by the final energy consumption of the enterprises, residents, and governments.

Fig 2 reveal that the coal mining industry contributes to 63.18%, the petroleum, coking and nuclear fuel industry 22.70%, the gas production and supply industry 10.49%, and the petroleum and natural gas exploitation industry 3.63% of the total carbon emissions. The results show that traditional fossil energy sources, that is the crude processing industries which are primarily coal and mining, are still dominant in the energy production of China. In contrast, the proportion of the deep processing of fossil energy sources (such as gas production and supply) is relatively small.

### Analysis of carbon-emission composition on the energy demand-side

It is noteworthy that the energy consumption on the demand-side comprises of intermediate consumption by enterprises, the final consumption by the residents and the government. This paper analyzes the carbon emissions of various sectors on the energy demand side in 2015, and the results are shown in Table 5. As can be seen from Table 5, the carbon emissions for intermediate demands accounted for 93.41% of the total emissions in 2015 in China, while the emissions for the final demands accounted for 6.59%. Therefore, the carbon emissions reduction on the energy demand-side should focus on the intermediate demands, rather than the consumption of residents and governments.

**Table 5:** Carbon emissions for intermediate and final demands in 2015.

Type	Carbon emissions(10 kilo-tons)	Percentage
Carbon emission for intermediate demands	1055692.85	93.41%
Carbon emission for final demands	74505.273	6.59%

**Fig 3:**
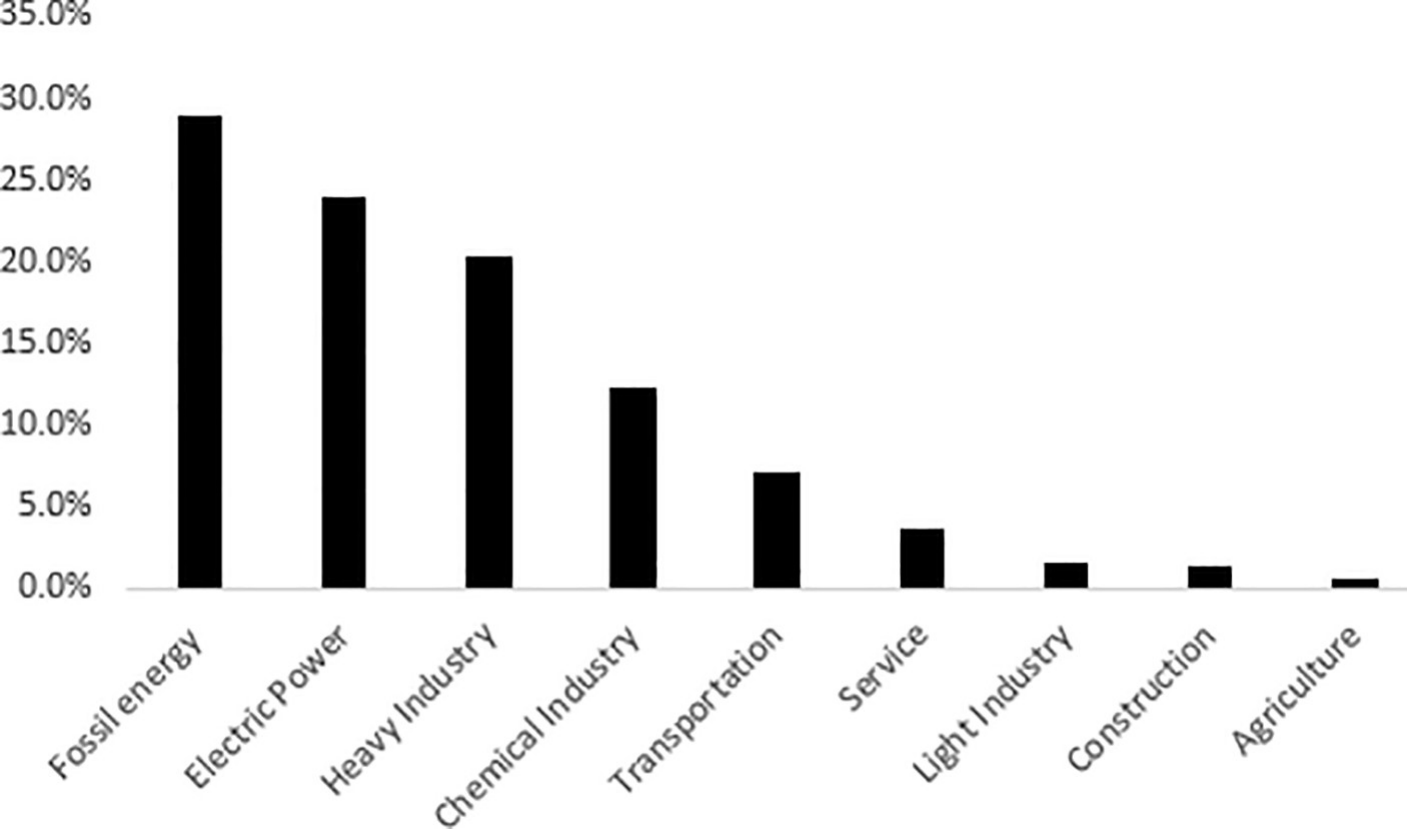
Proportion of carbon emissions in nine sectors.

According to the input-output statistics in 2015, China's industry is divided into 42 industries. If the carbon emissions of each industry are analyzed one by one, the layout is obviously insufficient. In order to analyze the carbon emissions of various industries in China concisely, this paper divides 42 sectors into 9 industrial sectors according to Jiang et al. [11] classification method. The carbon emissions from various sectors on the energy demand-side in the year 2015 were shown in Fig 3.

Fig 3 shows that the carbon emissions of fossil energy industry, power industry, heavy industry, chemical industry and transportation industry account for 28.87%, 23.96%, 20.39%, 12.27% and 7.23%, respectively. There are three reasons for the high carbon emission of the above industries: first, from the perspective of industrial structure, fossil energy industry, electric power industry, heavy industry, chemical industry and transportation industry account for a large proportion in the industrial structure. Second, from the analysis of energy structure, the power industry, fossil energy, chemical industry and transportation industry mainly use primary energy, such as thermal power generation, fossil energy, coal combustion, etc. Third, from the input factor analysis, heavy industry, electric power industry, fossil energy sector, chemical industry and transportation industry belong to high energy consumption industries, with large amount of energy input. In comparison, the carbon emissions of agriculture, construction, light industry and service industry are relatively small, accounting for 0.57%, 1.38%, 1.64% and 3.70% of the carbon emissions from intermediate use, respectively. The reason is that agriculture, construction, light industry and services mainly use electricity and less fossil energy.

**Fig 4:**
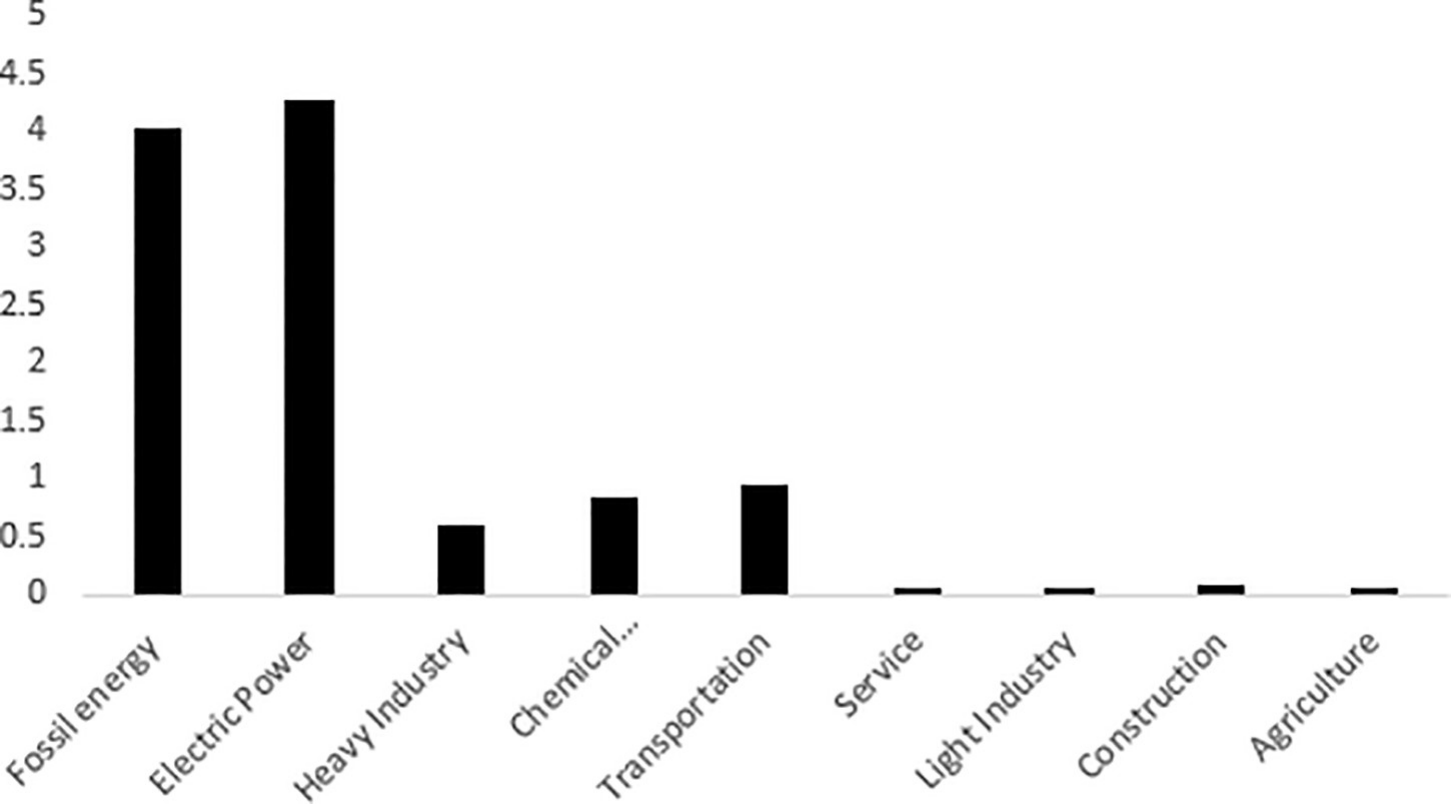
Carbon emission efficiencies of nine sectors.

In order to further analyze the characteristics of carbon emissions of various industries, this paper also analyzes the carbon emission efficiency of nine sectors. The unit of carbon emission efficiency in Fig 4 is tons/10,000RMB. Fig 4 shows that there are great differences in the carbon emission efficiency of various industries in China. Among them, the carbon emission efficiency of agriculture, light industry, service industry and construction industry is higher, while the carbon emission efficiency of power sector, fossil energy sector, transportation industry, chemical industry and heavy industry is relatively low, especially the power sector and fossil energy sector. The reason for the high carbon emission efficiency of light industry, agriculture, service industry and construction industry is that the energy input of these industries is mainly secondary energy such as electric power, while the input of high carbon energy such as coal is less. The power sector, fossil energy sector, transportation industry, chemical industry and heavy industry are energy input intensive industries, and the energy input is mainly fossil energy. For example, China's power industry mainly relies on coal-fired power generation, and the fossil energy sector mainly relies on high-carbon coal-fired energy. As a result, these industries produce more carbon emissions per unit of output, resulting in lower carbon efficiency.

## Conclusions

The factors of energy invest in gross capital formation and export into consideration, this work examined the carbon emission structures on the energy supply and demand-side of China in 2015 by using the energy consumption method and input-output method. The results showed that the ignorance of energy invest in gross capital formation and export led to the underestimation of carbon emissions for intermediate demands and overestimation of carbon emissions for final demands. On the energy supply-side, the carbon emissions from carbon-rich energy sources accounted for more than 70% of the total carbon emissions. The carbon emissions from natural gas, accounted for 3.45% of the total, indicating that the carbon emission could be optimized. On the energy demand-side, fossil energy, electrical power, heavy industry, chemical industry, and transportation sectors exhibited larger carbon emissions and lower carbon emission efficiencies. At the same time, agriculture, construction, light and service industries exhibited smaller amount of carbon emissions and higher carbon emission efficiencies, thereby showing the optimization of carbon emissions in China on the energy demand-side is dependent on the industrial structure.

This paper proposes the following policy recommendations. First, we should actively promote the research of carbon emission measurement. The results show that ignoring the impact of invest in gross capital formation and export on carbon emissions may lead to errors in the calculation results. Therefore, when calculating the carbon emissions of various industries on the demand side, the above factors must be taken into account to improve the accuracy of carbon emission measurement. Second, we need to optimize the mix of energy consumption. The conclusion of this paper shows that China has a lot of room to reduce carbon emissions in the future by optimizing the energy consumption structure. Specifically, on the premise of maintaining the demand for economic development, we should gradually increase the proportion of consumption of low-carbon energy such as natural gas through policy support and guidance, so as to reduce the proportion of consumption of high-carbon energy such as raw coal. Third, we need to optimize the industrial structure to save energy and reduce emissions. The research results of this paper show that the carbon emission efficiency of light industry, agriculture, service industry and construction industry is higher. The power sector, fossil energy, transportation, chemical and heavy industries are less carbon efficient. Under the political bidding system, local governments tend to develop manufacturing industries with high energy consumption and high pollution in order to win the competition from other local governments. Hence, in order to control carbon emissions from energy consumption, the central government should adjust the assessment and selection mechanism of local government officials, increase the weight of people's livelihood and environmental assessment content, reduce the negative impact of local governments on industrial structure upgrading, and achieve sustainable economic development.

## Supporting information

S1 Data (XLSX)
